# Gait stability and variability measures show effects of impaired cognition and dual tasking in frail people

**DOI:** 10.1186/1743-0003-8-2

**Published:** 2011-01-17

**Authors:** Claudine J Lamoth, Floor J van Deudekom, Jos P van Campen, Bregje A Appels, Oscar J de Vries, Mirjam Pijnappels

**Affiliations:** 1 Center for Human Movement Sciences, University Medical Centre Groningen, University of Groningen, the Netherlands; 2 Department of Geriatric Medicine, Slotervaart Hospital, Amsterdam, the Netherlands; 3 Medical Psychology, Slotervaart Hospital, Amsterdam, the Netherlands; 4 Department of Internal Medicine, VU University Medical Center, Amsterdam, the Netherlands; 5 Research Institute MOVE, Faculty of Human Movement Sciences, VU University Amsterdam, the Netherlands

## Abstract

**Background:**

Falls in frail elderly are a common problem with a rising incidence. Gait and postural instability are major risk factors for falling, particularly in geriatric patients. As walking requires attention, cognitive impairments are likely to contribute to an increased fall risk. An objective quantification of gait and balance ability is required to identify persons with a high tendency to fall. Recent studies have shown that stride variability is increased in elderly and under dual task condition and might be more sensitive to detect fall risk than walking speed. In the present study we complemented stride related measures with measures that quantify trunk movement patterns as indicators of dynamic balance ability during walking. The aim of the study was to quantify the effect of impaired cognition and dual tasking on gait variability and stability in geriatric patients.

**Methods:**

Thirteen elderly with dementia (mean age: 82.6 ± 4.3 years) and thirteen without dementia (79.4 ± 5.55) recruited from a geriatric day clinic, walked at self-selected speed with and without performing a verbal dual task. The Mini Mental State Examination and the Seven Minute Screen were administered. Trunk accelerations were measured with an accelerometer. In addition to walking speed, mean, and variability of stride times, gait stability was quantified using stochastic dynamical measures, namely regularity (sample entropy, long range correlations) and local stability exponents of trunk accelerations.

**Results:**

Dual tasking significantly (p < 0.05) decreased walking speed, while stride time variability increased, and stability and regularity of lateral trunk accelerations decreased. Cognitively impaired elderly showed significantly (p < 0.05) more changes in gait variability than cognitive intact elderly. Differences in dynamic parameters between groups were more discerned under dual task conditions.

**Conclusions:**

The observed trunk adaptations were a consistent instability factor. These results support the concept that changes in cognitive functions contribute to changes in the variability and stability of the gait pattern. Walking under dual task conditions and quantifying gait using dynamical parameters can improve detecting walking disorders and might help to identify those elderly who are able to adapt walking ability and those who are not and thus are at greater risk for falling.

## Background

One in three community-dwelling persons over 65 years of age falls at least once a year and this rate increases rapidly with age, and frailty [1]. Gait and balance disorders are suggested to better predict imminent falls than risk factors in other domains such as impaired vision and medication [1,2]. Therefore, the objective quantification of gait and balance disorders to detect persons who have high risk of falls is of utmost importance, especially in geriatric patients with cognitive decline who have a high tendency to fall.

Age-associated changes in gait characteristics, such as lower walking speed, reduced step length and increased step time have been interpreted as a more cautious, conservative gait pattern adopted to increase gait stability and decrease fall risk [3,4]. A more conscious gait pattern, however, may require more cognitive control and result in an attention demanding form of locomotion. If walking requires more cognitive control and becomes less automated, it might be more prone to be influenced by concurrent (cognitive) dual tasks. Even in healthy persons, dual tasks have been shown to affect walking performance [5,6]. With aging or pathologic conditions, gait changes in response to dual tasking might have a destabilizing effect on the gait pattern [7-9].

There is growing evidence that executive functions, plays an important role in the ability to perform a motor and cognitive task simultaneously in elderly [10-12]. Particularly in frail elderly and in persons with Alzheimer's disease, performance of a cognitive task during a motor task is reported to be associated with changes in gait stability and increased fall risk [10,13,14]. Stability is a significant component of standing balance and walking. A relatively new approach to quantify gait and balance stability is by means of time dependent analyses of variability using measures derived from the theory of stochastic dynamics [13,15-17]. In contrast to more conventional measures, (e.g. mean stride time, walking velocity), which in the case of cyclic movements treat each cycle as being an independent event unrelated to previous or subsequent strides, the applied methods assess fluctuations throughout the gait cycle, and as such provide insight into how behaviour unfolds, taking into account previous states of the system (e.g., cycle trajectory). Applying more traditional measures may mask the temporal variations of the gait pattern due to averaging procedures. A variety of dynamic measures has been used to quantify these time dependent variations in gait patterns, including Detrended Fluctuations Analysis [17], Sample Entropy [18], and Lyapunov exponents [19]. Although conceptually different, these measures assume that walking ability is reflected in dynamic characteristics, in terms of variability in, or local stability of gait patterns. The outcome variables obtained from studies using these methods, have proven to be sensitive to differences between various patient groups and between conditions and are suggested to be related to increased fall risk [4,9,19-22]. Hence, these dynamic parameters may have more power to differentiate between groups and to screen for high risk fallers, particularly in frail elderly whose fall risk might be enhanced by a cognitive impairment. We complemented the stride related measures with measures that quantify time varying patterns of trunk movements during walking and that are closely related to dynamic balance control during walking and standing. The aim of the present study was to examine gait stability and variability of geriatric patients with and without cognitive impairment under normal and dual task walking conditions. Based on previous studies, showing increased stride-to-stride variability during dual tasking and in elderly [4,14,22,23], and in line with the theoretical concept that health is characterized by 'organized' variability, while disease is defined by changes in the structure of variability [24], we hypothesized that dual tasking induced changes in the structure of the variability and decreased local stability of trunk acceleration patterns. Moreover, we anticipated that frail elderly patients with cognitive impairment would be more affected in their capacity to divide attention between a cognitive and a motor task simultaneously, resulting in less stable and more variable gait coordination than cognitive intact frail elderly patients.

## Methods

### Participants

Twenty six elderly were recruited on the geriatric day clinic of the hospital Slotervaart in Amsterdam. See Table 1 for the population characteristics. Subjects were included if they were 70 years of age or older and able to walk inside without an assistive device. Participants with a mobility impairment based on neurological or orthopaedic disorders limiting one or both legs were excluded as well as participants who did not understand the instructions. The IADL (Instrumental Activities of Daily living [25] was administered to assess dependency in daily life and the CCI (Charlson Comorbidity Index) [26] was determined to index the presence of co-morbidity in this geriatric group of patients. In all participants, the Mini Mental State Examination (MMSE) [27] and the Seven Minute Screen (SMS) [28] were administered. Participants were divided into two groups, one group suffering from cognitive impairment (MMSE < 23 and with a clinical diagnosis of Alzheimer's disease according to the criteria of the Alzheimer's Association , N = 13) and one group of cognitively unimpaired elderly (MMSE > 26; N = 13) [29]. Both groups differed significantly with respect to SMS scores with exception of the clock drawing subtest, and the IADL score, and not with respect to the CCI index (Table 1). The study was approved by the Medical Ethical Committee of the Slotervaart Hospital. Written informed consent was obtained from the participant and/or the caretaker (or legal attorney).

**Table 1:** Population characteristics, cognitive and activity of daily living test scores

	whole group	cognitive intact	cognitive impaired	group differences*	
	
	N = 26	N = 13	N = 13	z-value	p-value
Men/women (n)	10/16	6/7	4/9		
Age (y)	81.00 ± 5.13	79.38 ± 5.55	82.62 ± 4.29	1.31	0.19
Length (cm)	165.17 ± 9.10	166.00 ± 8.05	164.35 ± 11.75	0.59	0.55
Weight (kg)	67.52 ± 12.90	72.59 ± 11.97	62.45 ± 12.16	2.18	0.03
MMSE	23.12 ± 5.81	28.23 ± 1.09	18.00 ± 3.54	4.36	< 0.001
SMS	61.74 ± 109.73	-2.13 ± 15.91	125.62 ±125.81	3.82	< 0.001
BTO	17.65 ± 31.04	1.00 ± 3.61	34.31 ± 37.32	3.45	0.001
ECR	9.73 ± 9.73	12.62 ± 3.15	6.85 ± 4.41	3.11	0.002
CD	9.00 ± 3.43	10.00 ± 2.35	8.01 ± 4.10	1.17	0.243
VF	10.19 ± 3.95	12.54 ± 3.02	7.85 ± 3.39	3.40	< 0.001
IADL	4.69 ± 5.04	7.54 ± 5.29	1.85 ± 2.73	2.89	0.003
CCI	2.00 ± 1.26	2.15 ± 1.34	1.85 ± 1.23	0.62	0.58

Values are mean ± standard deviations. Statistical differences between the cognitive intact and cognitive impaired participants are indicated by z- and p-values (based on Mann-Whitney test). Abbreviations: MMSE = Minimal Mental Scale examination; Range: 0-30, scores < 23 indicating cognitive impairment. SMS = Seven Minute Screening test, higher values indicate cognitive impairment, low or negative values the absence of cognitive impairment. BTO = The Benton Temporal Orientation; Range: 0 = intact orientation 113 = severe disorientation; ECR = Enhanced Cued Recall, Range: 0-16; CD = Clock drawing, maximum score = 14; VF = Verbal Fluency task, range: 0-45.; IADL = Instrumental Activities of Daily living, maximal dependency = score of 14; CCI = Charlson Comorbidity Index.

### Procedure

Participants walked for 3 minutes (about 160 m) in a well-lit, empty 40 m long corridor at self-selected speed. Walking was performed once without and once while performing a verbal dual task. In the dual task condition, participants were asked to perform a letter fluency task in which the subject had to name as many words starting with a predefined letter "R" or "G"[30]. This task relies on set-shifting and speed of processing which is considered an executive function. During walking with the dual task, participants were instructed not to prioritize either one of the tasks. Participants performed the task also seated during three minutes. The number of different words was counted.

During walking trials, trunk accelerations in 3 orthogonal directions were measured with a tri-axial ambulant accelerometer (64×64×13 mm; DynaPort^® ^MiniMod, McRoberts BV, The Hague, the Netherlands), fixed with an elastic belt at the level of third lumbar spine segment close to the centre of mass [31]. Sample frequency was 100 Hz.

### Data analysis

Anterior-posterior and medio-lateral acceleration time series were analyzed. All time series were corrected for horizontal tilt and low pass filtered with a 3^th ^order Butterworth filter with a cut-off frequency of 20 Hz. From the anterior-posterior acceleration signal, time indices of left and right foot contacts were determined. From these foot contact moments stride times were calculated by subtracting subsequent foot contact times of the same foot. For all participants and conditions, at least 150 successive strides (leaving start and end steps out) were included in all analyses, however bends in the circuit, were removed from the data using a median filter [32]

For each participant and condition, walking speed, mean and coefficient of variation (CV) of stride times were calculated. Stride frequency was defined as the inverse of the mean left and right stride time intervals. Phase variability index (PVI) was calculated, based on the mean and variability of relative phases between consecutive contralateral foot contacts [33]. Lower PVI values represent more consistent timing and gait symmetry.

For medio-lateral and anterior-posterior trunk accelerations, the magnitudes of the time series were calculated as the root mean squares (RMS) and peak accelerations within strides were determined. In addition, time dependent variations of stride variables and trunk acceleration patterns were calculated. Specifically, the structure of stride variability (stride-to-stride variability) and trunk accelerations patterns were assessed as indicators of dynamic balance ability during walking, using the scaling exponent α (DFA) [34], the local stability exponent (LSE)[35] and the sample entropy (SEn) [18], which are briefly described below. For a mathematical explanation see the associated references and for applications see references [16,36].

Perturbations of stability do not inevitably only come from outside, even during unimpeded walking, 'small scale' perturbations created by neuromuscular noise [37] continuously perturb the locomotor system. These perturbations may manifest themselves as the natural variations exhibited during walking, for instance in the stride-to-stride variability or in terms of changes in so-called local stability. Whereas the standard deviation or coefficient of variation of stride times provide information about the magnitude of stride variability, the extent to which stride interval time series exhibited long range correlations (i.e. similar patterns of variation across multiple time scales) is quantified by the α of Detrended Fluctuations Analysis (DFA). Before applying DFA, outliers in the stride time data, caused by the turns in the circuit, were removed from the data using a median filter[32]. If the outcome variable α is between 0.5 and 1, this indicates the presence of long range correlations in the time series, i.e. future fluctuations are better predicted by past fluctuation and accordingly indicate a stable more structured pattern if α get near 1. For uncorrelated time-series (e.g. white noise) α = 0.5. When 0 < α < 0.5 a different type of power-law correlation exist such that large and small values of the time-series are likely to alternate. When α increases above 1 to 1.5, behaviour is no longer determined by power law. DFA was applied to stride time, as well as to medio-lateral and anterior-posterior trunk accelerations.

The ability to resist perturbations was assessed by means of maximum finite time lyapunov exponents or so called local stability exponents (LSE) [35]. The size of the LSE quantifies the average rate of divergence of initially nearby trajectories in state space over a specified finite time interval. In a stable system, nearby trajectories will converge with time, whereas in an unstable system initially nearby trajectories will diverge with time [35]. When a LSE is negative, any perturbation in the gait pattern will exponentially damp out and initially nearby trajectories remain close. In contrast, for larger LSE values, nearby points diverge as time evolves and produce instability. The time delay estimated was 10% of the gait cycle for all reconstructed state spaces. Following previous studies, an embedding dimension of 5 was chosen, since this has been proven to be appropriate for kinematic gait data. Δt =1 -3 strides. As average stride times were different for participants walking with different speeds, the time axes for the LSE curves of trunk acceleration were rescaled per trial by multiplying by the average stride frequency [38].

The degree of predictability or repeatable pattern features in acceleration time series was indexed by means of the SEn [18]. A periodic time series is completely predictable and will have a SEn of zero. SEn is defined as the negative natural logarithm of an estimate of the conditional probability of epochs of length *m *(in this study *m *= 5) that match point-wise within a tolerance *r *and repeats itself for m+1 points. Small SEn values are associated with great regularity while large SEn values represent a small chance of similar data being repeated. The data were first normalized to unit variance, rendering the outcome scale-independent. Software available at PhysioNet was used to calculate SEn[39].

### Statistical analysis

Statistical analysis was performed using SPSS version 14.0. Level of significance was set at p < 0.05. Non-parametric statistics was applied since normality assumptions were not met for most of the outcome variables. Group effect and main condition effects were tested for significance using the Mann-Whitney test and Wilcoxon signed rank test. To examine the relation between SMS, MMSE scores and gait and trunk variables, Spearman correlations were calculated.

## Results

### Condition effects

The number of enumerating words did not differ significantly (z = 0.12; *p *= 0.91) between dual (walking; 15.6 ± 4.5) and single task (sitting; 16.0 ± 8.3).

Walking speed and stride frequency decreased significantly under the dual task condition, while stride-to-stride variability increased (α decreased), mean stride time, CV of stride times, and the PVI increased significantly (Table 2).

**Table 2:** Effect of dual tasking on gait variables.

Variables	Walking	Dual Tasking	z- value	p
				
speed (m/sec)	0.92 ± 0.24	0.80 ± 0.21	4.31	< 0.001
stride frequency (strides/sec)	0.82 ± 0.11	0.77 ± 0.11	3.95	< 0.001
mean stride time (sec)	1.23 ± 0.18	1.33 ± 0.17	3.87	< 0.001
CV stride time (%)	3.61 ± 2.30	4.41 ± 2.34	2.83	0.005
PVI (%)	15.08 ± 7.60	17.68 ± 8.49	3.54	< 0.001
α stride times	0.85 ± 0.14	0.77 ± 0.15	2.48	0.013

Values during walking and dual tasking for: walking speed, stride frequency, mean and coefficient of variation (CV) of stride times, the phase variability index (PVI) and stride-to-stride variability (α). Values are mean ± standard deviations. Statistical differences between conditions are indicated by z- and *p*-values (based on Wilcoxon signed rank test).

**Figure 1:**
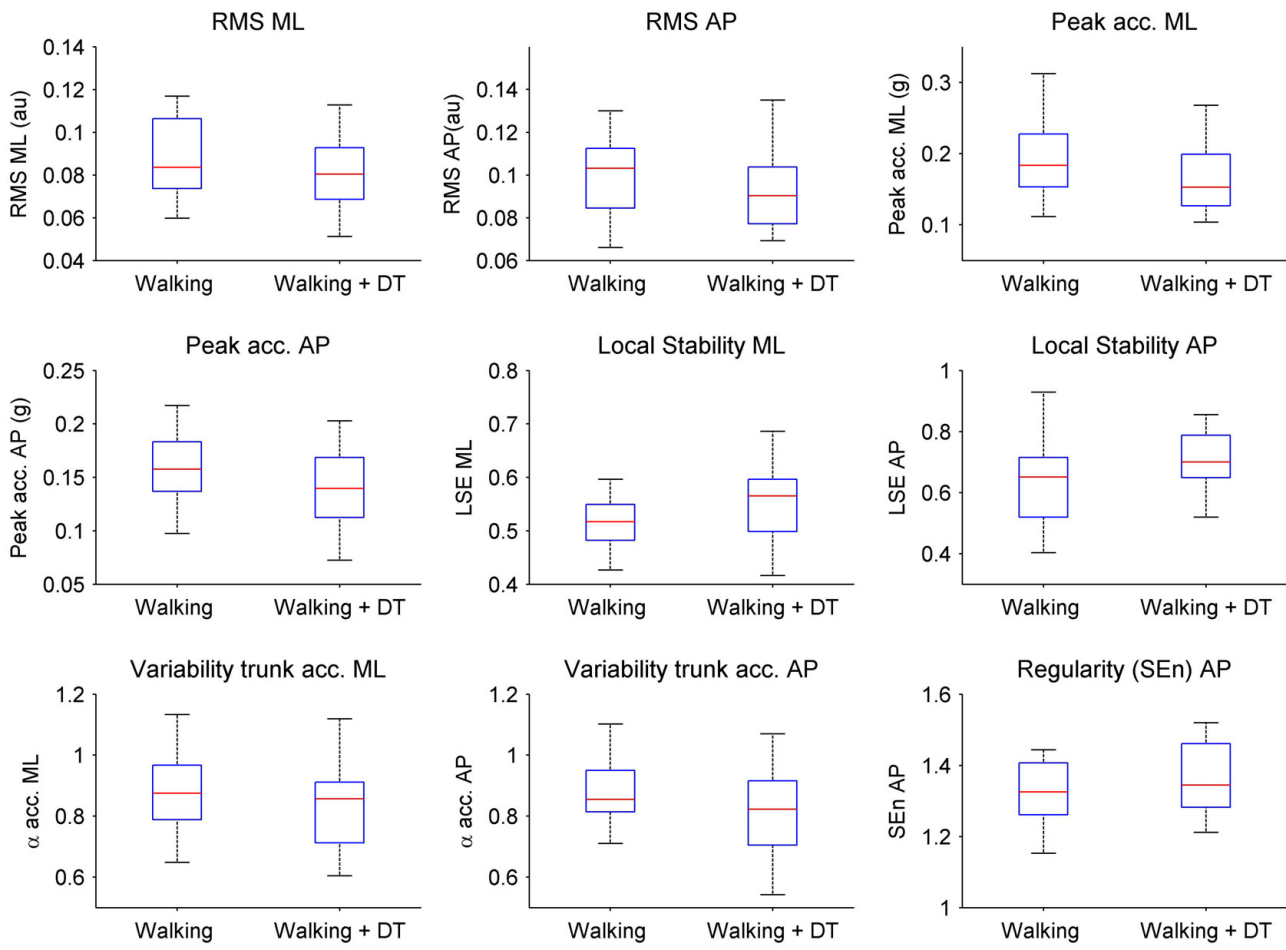
**Effect of dual tasking**. Boxplots of significant (all p < 0.05) effects of dual tasking on medio-lateral (ML) and anterior-posterior (AP) trunk accelerations patterns. The lower and upper lines of the box are the 25th and 75th percentiles of the sample. The line in the middle of the box is the sample median. The vertical lines extending above and below the box show the extent of the rest of the sample

During dual tasking, the RMS and peak values of anterior-posterior and medio-lateral trunk accelerations, as well as stride-to-stride variability (α) were significantly lower (all *p *< 0.001) compared to normal walking, whereas the LSE in anterior-posterior and medio-lateral trunk accelerations were significantly (*p *< 0.001) increased, indicating decreased stability (Figure 1). Dual tasking further significantly decreased the regularity as indicated by a larger SEn of anterior-posterior trunk accelerations (*p *= 0.03) but not of medio-lateral accelerations.

### Group effect

No significant difference in the number of enumerating words during walking was found between cognitively intact and cognitively impaired elderly (14.4 ± 1.2 vs. 16.8 ± 1.4, respectively; *p *= 0.19), indicating that all participants could perform the task.

**Figure 2:**
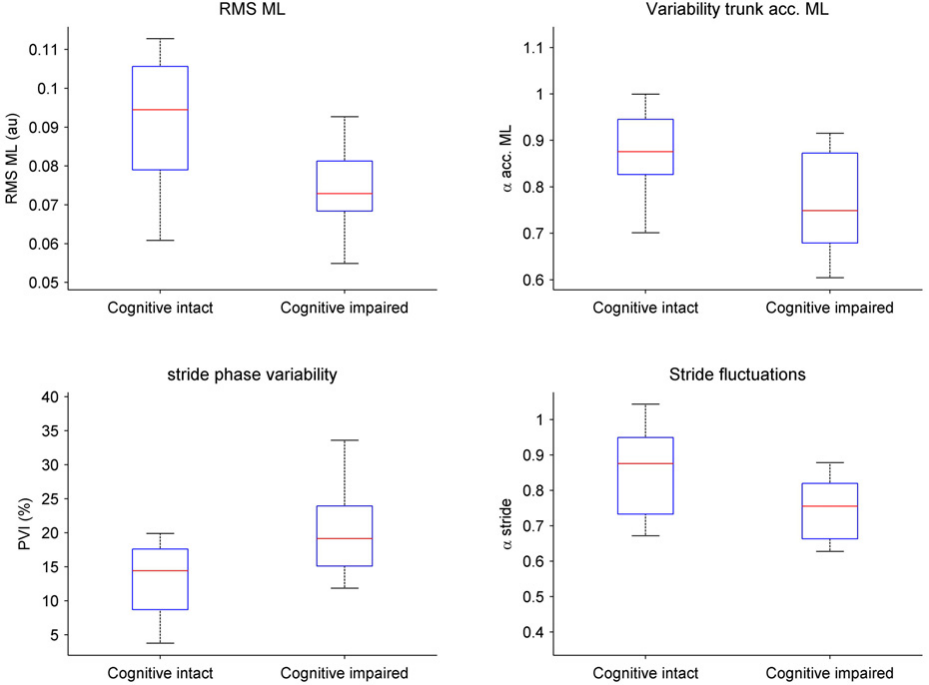
**Group differences**. Boxplots of significant (all p < 0.05) differences between the cognitive impaired and cognitive intact elderly on trunk variability of ML trunk acceleration patterns as quantified by the RMS and the α and of stride-to-stride variability quantified by the phase variability index (PVI) and the α of the stride-to-stride fluctuations. The lower and upper lines of the box are the 25th and 75th percentiles of the sample. The line in the middle of the box is the sample median. The vertical lines extending above and below the box show the extent of the rest of the data.

For walking without dual tasking, no significant group differences were found for any of the gait or trunk variables. However, when walking while performing a dual task, significant differences were observed for the RMS of the medio-lateral trunk accelerations (z = 1.97, *p *= 0.04), the structure of variability (α) of the medio-lateral trunk accelerations (z = 2.64, *p *= 0.008), and for trunk anterior-posterior peak accelerations (z = 1.92, *p = *0.05). Lower values of α for medio-lateral trunk accelerations in the cognitive impaired elderly indicated a less correlated (more random) trunk acceleration pattern than in the cognitive intact group. In addition, significant group effects were observed for PVI (z = -2.18, *p *= 0.03) and stride-to-stride variability (z = -2.13, *p *= 0.03), both implying an increased variability of gait timing in the cognitive impaired elderly (Figure 2). In contrast, walking velocity and mean and CV of stride times were not significantly different between groups (Table 3).

**Table 3:** Group effect on gait variables.

Variables		Cognitive intact	Cognitive impaired	z- value	p
					
speed (m/sec)	Walk	0.95 ± 0.21	0.88 ± 0.27	0.404	ns
	Walk +DT	0.78 ± 0.24	0.83 ± 0.20	0.293	ns
stride frequency	Walk	0.84 ± 0.09	0.80 ± 0.12	0.686	ns
(strides/sec)	Walk +DT	0.77 ± 0.12	0.78 ± 0.10	0.692	ns
mean stride time (sec)	Walk	1.20 ± 1.44	1.27 ± 0.19	0.564	ns
	Walk +DT	1.32 ± 0.19	1.29 ± 0.16	0.692	ns
CV stride time (%)	Walk	2.95 ± 1.77	4.20 ± 2.70	0.564	ns
	Walk +DT	5.00 ± 2.67	3.67 ± 1.67	1.20	ns
PVI (%)	Walk	11.29 ± 7.43	15.87 ± 5.89	1.32	ns
	Walk +DT	20.84 ± 8.51	14.32 ± 8.02	2.18	0.03
α stride times	Walk	0.87 ± 0.15	0.84 ± 0.16	0.86	ns
	Walk +DT	0.74 ± 0.15	0.84 ± 0.11	2.23	0.03

Values for the cognitive impaired (CI) and cognitive intact elderly for" walking speed, stride frequency, mean and coefficient of variation (CV) of stride times, the phase variability index (PVI) and stride-to-stride variability (α).Values are mean ± standard deviations. Statistical differences between conditions are indicated by z- and *p*-values (based on Mann-Whitney U test).

Overall, correlations between MMSE, SMS scores, and stride and trunk acceleration measures were low (r < 0.3). Within the cognitive impaired group, the associations were higher for several gait measures (see Table 4). Of the SMS tests, the temporal orientation and verbal fluency subtests correlated moderately to high (range 0.5-0.7) with the gait variables, whereas no association was found for the clock drawing and enhanced cued recall subtests.

**Table 4:** Association between cognitive function and gait variables during dual tasking

		speed	mean ST	PVI	CV stride times	α strides	RMS anterior-posterior	LSE anterior-posterior	Peak acc. m
Cognitive impaired	MMSE	0.70**	-0.72**	-0.68**	-0.68**	0.32	-0.56*	-0.74**	0.62*
	SMS	-0.66**	0.57*	0.67**	0.37	-0.56*	0.50*	0.43	-0.63*
Cognitive intact	MMSE	0.08	-0.36	0.14	-0.14	-0.17	-0.26	0.61*	-0.19
	SMS	0.10	0.29	-0.28	0.36	-0.28	0.38	0.19	-0.30

**P < 0.01; * P < 0.05; Spearman correlations

Correlations between the mini mental state examination (MMSE) scores, the Seven Minute Screening (SMS) test scores and walking speed, mean, coefficient of variation (CV), phase variability index (PVI) and long range correlations of stride times , the root means square (RMS) and local stability exponent (LSE) of anterior-posterior accelerations and the medio-lateral peak acceleration.

## Discussion

The goal of the present study was to assess the effects of dual tasking on gait stability and variability of frail elderly with and without cognitive impairment. We expected that the effect of dual tasking and differences between the cognitive impaired and cognitive intact frail elderly would reveal in the structure of variability of medio-lateral and anterior-posterior trunk accelerations and in stride variability measures, rather than in general velocity and stride time variables.

In general, all participants altered their gait pattern in response to dual tasking by decreasing walking speed and increasing stride time. However, despite lower walking speed, trunk accelerations patterns were more irregular and variable and local stability was decreased in the dual task condition. In addition, stride variability was increased and less structured as quantified by the larger PVI and a decline in the measure α of the DFA. Thus, although the slowing of gait while performing a dual task could reflect an adaptation to more difficult circumstances, the resulting trunk adaptations showed a consistent and therefore statistically significant instability factor, possibly leading to an increased fall risk. These results support the notion that gait is not merely an automated motor activity, but utilizes higher levels of cognitive input, particularly in this population of frail elderly.

Interestingly, no significant differences between the cognitive intact and cognitive impaired elderly were found in gait variables under single task condition. In line with the findings of the study of Toulotte et al.[40], who found differences in gait variables between fallers and non-fallers only in dual task conditions, dual tasking appears to affect walking stronger in de cognitive impaired elderly than in the non-cognitive elderly. This could also explain why over the whole group no significant correlation was observed between the cognitive scores and scores on the test indicative for executive function (clock drawing) and gait variables. However, within the cognitively impaired group, significant associations between cognitive function and several variability measures were found. Walking speed was strongly related to the MMSE and total SMS scores for the cognitive impaired subject but not for the cognitive intact subjects. Moreover walking speed did not discriminate between cognitively impaired and intact participants. This can be explained by the ceiling effect on the MMSE values for the cognitive intact subjects, that is the MMSE values show very little between subject variation, while between subject variation in walking speed is about similar in both groups. In line with our findings, other studies reported weak associations between executive function and/or attention and gait variables under standard walking conditions, that became stronger when walking while performing a dual task [9,41,42]. This association, however, was not observed in healthy subjects. In patients without cognitive impairment however, executive function is found to be independently associated with gait function [42].

So, the relation between cognitive functioning and gait variability becomes more visible when the task is more challenging and with a gait pattern that is already impaired, such as in the frail elderly or in patients with Alzheimer's disease. Simultaneously, executed attention demanding tasks compete for attention. In contrast to healthy young and elderly people, who in such situations give priority to the walking task at the cost of lower performance on the cognitive task [43], the stability and variability of the gait pattern deteriorated for all our patients while the quality of the cognitive task was similar in the sitting and walking condition. It is unclear why our subjects prioritise the cognitive task (no such instructions were given), but the same findings have been reported for different patient groups and the favour of one activity over the other might depend on task complexity [7,44].

In line with previous studies, we found that measures of stride variability and consistency were more sensitive to detect gait changes due to dual tasking than more global gait measures such as gait speed [9,44]. We complemented these stride related measures with trunk measures that are closely related to dynamic balance control during walking and standing. Presumably, the stability and the pattern of variability of the trunk movements is indicative of the adaptability and the ability to react adequately to withstand small perturbations [7,16]. The results signify that a more detailed knowledge on gait coordination acquired from this type of analyses might help to identify those who are able to adapt walking ability and those who are not and are thus at greater risk for falls.

Clinically, fall risk is currently quantified mainly by counting the number of falls over a specific time span, for example by using a fall-diary. However, this is a time consuming method and has proven to be not reliable especially in patients who are forgetful [45]. Moreover, falling is an extreme symptom of loss of balance, and one would like to detect the risk of falling in an earlier phase. Although no direct clinical conclusions can be drawn with respect to the detection of falls, our results point out that a combination of accelerometry and off-line dynamical analysis to quantify stride as well as trunk variability and stability provides an objective instrument for screening persons at high risk. Hence, it can be a diagnostic tool for the clinician to examine gait ability and associated fall-risk. Notwithstanding the immediate benefits of accelerometric systems for clinical purposes (i.e., compact and easy-to-use, the subject's minimal awareness of the measuring process on the part of the subject), they also have several drawbacks such as the need to pre-process the data, and the translation to clinically applicable outcome measures. These processes are being automated and simplified for clinical use.

A limitation of the present study is that the groups were small. We nevertheless found significant differences due to dual tasking and between groups. Furthermore, the cognitive intact elderly of our study attended the diagnostic geriatric outpatient clinic for multiple problems, and used multiple medications. Therefore, we did apply a post-hoc analysis with covariate medications but found no significant differential effect between both groups.

## Conclusions

In conclusion, the results of the present study provide further support that changes in cognitive functioning are likely to contribute to an increased fall risk, especially in frail elderly when tasks such as walking requires more attention and are combined with concurrent (cognitive) tasks. Walking under dual-task conditions could therefore be helpful when screening individuals with gait impairments and those at risk for falling, as this appears to unmask gait impairments that can provoke falls. We further showed that these impairments can be best discerned by variability and stability measures. When noticing gait instability, future falls might be prevented, by early intervention focusing on fall prevention.

## Competing interests

The authors, Claudine Lamoth, Floor JA van Deudekom, Jos P van Campen, Bregje A. Appels, Oscar J de Vries, and, Mirjam Pijnappels declare that they have no proprietary, financial, professional, or other personal competing interests of any nature or kind.

## Authors' contributions

CJL was involved in the conception of the research project, design, analysis and interpretation of the data analysis and writing of the manuscript. FJD was involved in the design and organization of the study and the acquisition of the data. JPC contributed to the conception and organization of the study and revising the manuscript. BA was involved in the organization of the study and the acquisition of the data. OJV participated in the conception and organization of the study and revising the manuscript. MP was involved in the design of the study, and revising the manuscript. All authors read and approved the manuscript.

## References

[B1] Liu-Ambrose TY Ashe MC Graf P Beattie BL Khan KM Increased risk of falling in older community-dwelling women with mild cognitive impairment Phys Ther 2008 88 1482 1491 10.2522/ptj.20080117 18820094 PMC3514550

[B2] Ganz DA Bao Y Shekelle PG Rubenstein LZ Will my patient fall? Jama 2007 297 77 86 10.1001/jama.297.1.77 17200478

[B3] Menz HB Lord SR Fitzpatrick RC Age-related differences in walking stability Age Ageing 2003 32 137 142 10.1093/ageing/32.2.137 12615555

[B4] Buzzi UH Stergiou N Kurz MJ Hageman PA Heidel J Nonlinear dynamics indicates aging affects variability during gait Clin Biomech 2003 18 435 443 10.1016/S0268-0033(03)00029-9 12763440

[B5] Beauchet O Dubost V François R Herrmann R Kressig W Stride-to-stride variability while backward counting among healthy young adults J Neuroeng Rehabil 2005 2 26 10.1186/1743-0003-2-26 16095533 PMC1190208

[B6] Lamoth C Roerdink M Beek P Acoustically-paced treadmill walking requires more attention than unpaeed treadmill walking in healthy young adults Gait Posture 2007 26 S96

[B7] Lamoth CJ Stins JF Pont M Kerckhoff F Beek PJ Effects of attention on the control of locomotion in individuals with chronic low back pain J Neuroeng Rehabil 2008 5 13 10.1186/1743-0003-5-13 18439264 PMC2387160

[B8] Lindenberger U Marsiske M Baltes PB Memorizing while walking: increase in dual-task costs from young adulthood to old age Psychol Aging 2000 15 417 436 10.1037/0882-7974.15.3.417 11014706

[B9] Springer S Giladi N Peretz C Yogev G Simon ES Hausdorff JM Dual-tasking effects on gait variability: the role of aging, falls, and executive function Mov Disord 2006 21 950 957 10.1002/mds.20848 16541455

[B10] Sheridan PL Solomont J Kowall N Hausdorff JM Influence of executive function on locomotor function: divided attention increases gait variability in Alzheimer's disease J Am Geriatr Soc 2003 51 1633 1637 10.1046/j.1532-5415.2003.51516.x 14687395

[B11] Hausdorff JM Schweiger A Herman T Yogev-Seligmann G Giladi N Dual-task decrements in gait: contributing factors among healthy older adults J Gerontol A Biol Sci Med Sci 2008 63 1335 1343 19126846 10.1093/gerona/63.12.1335 PMC3181497

[B12] Siu KC Chou LS Mayr U van Donkelaar P Woollacott MH Attentional mechanisms contributing to balance constraints during gait: the effects of balance impairments Brain Res 2009 1248 59 67 10.1016/j.brainres.2008.10.078 19028462 PMC3133742

[B13] Allali G Assal F Kressig RW Dubost V Herrmann FR Beauchet O Impact of impaired executive function on gait stability Dement Geriatr Cogn Disord 2008 26 364 369 10.1159/000162358 18852489

[B14] Beauchet O Allali G Annweiler C Bridenbaugh S Assal F Kressig RW Herrmann FR Gait variability among healthy adults: low and high stride-to-stride variability are both a reflection of gait stability Gerontology 2009 55 702 706 10.1159/000235905 19713694

[B15] Kang HG Costa MD Priplata AA Starobinets OV Goldberger AL Peng CK Kiely DK Cupples LA Lipsitz LA Frailty and the degradation of complex balance dynamics during a dual-task protocol J Gerontol A Biol Sci Med Sci 2009 64 1304 1311 19679739 10.1093/gerona/glp113 PMC2781784

[B16] Lamoth C van Lummel RC Beek PJ Athletic skill level is reflected in body sway: a test case for accelometry in combination with stochastic dynamics Gait Posture 2009 29 546 551 10.1016/j.gaitpost.2008.12.006 19138522

[B17] Hausdorff JM Ashkenazy Y Peng CK Ivanov PC Stanley HE Goldberger AL When human walking becomes random walking: fractal analysis and modeling of gait rhythm fluctuations Physica A 2001 302 138 147 10.1016/S0378-4371(01)00460-5 12033228

[B18] Pincus SM Approximate entropy as a measure of system complexity Proceedings of the National Academy of Sciences of the United States of America 1991 88 2297 2301 10.1073/pnas.88.6.2297 11607165 PMC51218

[B19] Dingwell JB Cusumano JP Nonlinear time series analysis of normal and pathological human walking Chaos 2000 10 848 863 10.1063/1.1324008 12779434

[B20] Sekine M Akay M Tamura T Higashi Y Fujimoto T Fractal dynamics of body motion in patients with Parkinson's disease J Neural Eng 2004 1 8 15 10.1088/1741-2560/1/1/002 15876617

[B21] Hausdorff JM Rios DA Edelberg HK Gait variability and fall risk in community-living older adults: A 1-year prospective study Arch Phys Med Rehabil 2001 82 1050 1056 10.1053/apmr.2001.24893 11494184

[B22] Lockhart TE Liu J Differentiating fall-prone and healthy adults using local dynamic stability Ergonomics 2008 51 1860 1872 10.1080/00140130802567079 19034782 PMC2892176

[B23] Hausdorff JM Mitchell SL Firtion R Peng CK Cudkowicz ME Wei JY Goldberger AL Altered fractal dynamics of gait: reduced stride-interval correlations with aging and Huntington's disease J Appl Physiol 1997 82 262 269 9029225 10.1152/jappl.1997.82.1.262

[B24] Goldberger AL Amaral LA Hausdorff JM Ivanov P Peng CK Stanley HE Fractal dynamics in physiology: alterations with disease and aging Proceedings of the National Academy of Sciences of the United States of America 2002 99 Suppl 1 2466 2472 10.1073/pnas.012579499 11875196 PMC128562

[B25] Lawton MP Brody EM Assessment of older people: self maintaining of older people; self maintaining and instrumental activities in daily living The Gerontologist 1969 9 179 186 5349366

[B26] Charlson ME Pompei P Ales K CR M A new method of classifying prognostic comorbidity in longitudinal studies: development and validation J Chron Dis 1987 40 373 383 10.1016/0021-9681(87)90171-8 3558716

[B27] Folstein MF Folstein SE McHugh PR "Mini-mental state". A practical method for grading the cognitive state of patients for the clinician J Psychiatr Res 1975 12 189 198 10.1016/0022-3956(75)90026-6 1202204

[B28] Solomon PR Hirschoff A Kelly B Relin M Brush M DeVeaux RD Pendlebury WW A 7 minute neurocognitive screening battery highly sensitive to Alzheimer's disease Arch Neurol 1998 55 349 355 10.1001/archneur.55.3.349 9520009

[B29] Kim SY Caine ED Utility and limits of the mini mental state examination in evaluating consent capacity in Alzheimer's disease Psychiatric services 2002 53 Washington, DC 1322 1324 10.1176/appi.ps.53.10.1322 12364686

[B30] Nessler D Mecklinger A Penney TB Perceptual fluency, semantic familiarity and recognition-related familiarity: an electrophysiological exploration Brain research 2005 22 265 288 15653299 10.1016/j.cogbrainres.2004.03.023

[B31] Moe-Nilssen R Test-retest reliability of trunk accelerometry during standing and walking Arch Phys Med Rehabil 1998 79 1377 1385 10.1016/S0003-9993(98)90231-3 9821897

[B32] Hausdorff JM Lertratanakul A Cudkowicz ME Peterson AL Kalition D Goldberger AL Dynamic markers of altered gait rhythm in amyotrophic lateral sclerosis J Appl Physiol 2000 88 2045 2053 10846017 10.1152/jappl.2000.88.6.2045

[B33] Plotnik M Giladi N Hausdorff JM A new measure for quantifying the bilateral coordination of human gait: effects of aging and Parkinson's disease Exp Brain Res 2007 181 561 570 10.1007/s00221-007-0955-7 17503027

[B34] Peng CK Havlin S Stanley HE Goldberger AL Quantification of scaling exponents and crossover phenomena in nonstationary heartbeat time series Chaos 1995 5 82 87 10.1063/1.166141 11538314

[B35] Rosenstein MT Collins JJ De Luca CJ A practical method for calculating largest Lyapunov exponents from small data sets Physica D 1993 65 117 134 10.1016/0167-2789(93)90009-P

[B36] Roerdink M De Haart M Daffertshofer A Donker SF Geurts AC Beek PJ Dynamical structure of center-of-pressure trajectories in patients recovering from stroke Exp Brain Res 2006 174 256 269 10.1007/s00221-006-0441-7 16685508

[B37] Faisal AA Selen LP Wolpert DM Noise in the nervous system Nat Rev Neurosci 2008 9 292 303 10.1038/nrn2258 18319728 PMC2631351

[B38] Dingwell JB Marin LC Kinematic variability and local dynamic stability of upper body motions when walking at different speeds J Biomech 2006 39 444 452 16389084 10.1016/j.jbiomech.2004.12.014

[B39] Goldberger AL Amaral LA Glass L Hausdorff JM Ivanov PC Mark RG Mietus JE Moody GB Peng CK Stanley HE PhysioBank, PhysioToolkit, and PhysioNet: components of a new research resource for complex physiologic signals Circulation 2000 101 E215 220 10851218 10.1161/01.cir.101.23.e215

[B40] Toulotte C Thevenon A Watelain E Fabre C Identification of healthy elderly fallers and non-fallers by gait analysis under dual-task conditions Clin Rehabil 2006 20 269 276 10.1191/0269215506cr929oa 16634347

[B41] Yogev-Seligmann G Hausdorff JM Giladi N The role of executive function and attention in gait Mov Disord 2008 23 329 342 10.1002/mds.21720 18058946 PMC2535903

[B42] Ble A Volpato S Zuliani G Guralnik JM Bandinelli S Lauretani F Bartali B Maraldi C Fellin R Ferrucci L Executive function correlates with walking speed in older persons: the InCHIANTI study J Am Geriatr Soc 2005 53 410 415 10.1111/j.1532-5415.2005.53157.x 15743282

[B43] Li KZ Lindenberger U Freund AM Baltes PB Walking while memorizing: age-related differences in compensatory behavior Psychol Sci 2001 12 230 237 10.1111/1467-9280.00341 11437306

[B44] Dubost V Annweiler C Aminian K Najafi B Herrmann FR Beauchet O Stride-to-stride variability while enumerating animal names among healthy young adults: result of stride velocity or effect of attention-demanding task? Gait Posture 2008 27 138 143 10.1016/j.gaitpost.2007.03.011 17467275

[B45] Ganz DA Higashi T Rubenstein LZ Monitoring falls in cohort studies of community-dwelling older people: effect of the recall interval J Am Geriatr Soc 2005 53 2190 2194 10.1111/j.1532-5415.2005.00509.x 16398908

